# An evaluation of kurtosis beamforming in magnetoencephalography to localize the epileptogenic zone in drug resistant epilepsy patients

**DOI:** 10.1016/j.clinph.2017.12.040

**Published:** 2018-06

**Authors:** Michael B.H. Hall, Ida A. Nissen, Elisabeth C.W. van Straaten, Paul L. Furlong, Caroline Witton, Elaine Foley, Stefano Seri, Arjan Hillebrand

**Affiliations:** a Aston Brain Centre, School of Life and Health Sciences, Aston University, Birmingham B4 7ET, UK; b Department of Clinical Neurophysiology and MEG Center, Neuroscience Campus Amsterdam, VU University Medical Center, Postbus 7057, 1007 MB Amsterdam, The Netherlands; c Department of Clinical Neurophysiology and Paediatric Epilepsy Surgery Programme, The Birmingham Children's Hospital NHS Foundation Trust, Birmingham, UK

**Keywords:** MEG, magnetoencephalography, iEEG, intracranial EEG, ECD, equivalent current dipole, MRI, magnetic resonance imaging, EZ, epileptogenic zone, tSSS, temporal signal space separation, Epilepsy, MEG, Kurtosis, Beamforming, Neuroimaging

## Abstract

**Objective:**

Kurtosis beamforming is a useful technique for analysing magnetoencephalograpy (MEG) data containing epileptic spikes. However, the implementation varies and few studies measure concordance with subsequently resected areas. We evaluated kurtosis beamforming as a means of localizing spikes in drug-resistant epilepsy patients.

**Methods:**

We retrospectively applied kurtosis beamforming to MEG recordings of 22 epilepsy patients that had previously been analysed using equivalent current dipole (ECD) fitting. Virtual electrodes were placed in the kurtosis volumetric peaks and visually inspected to select a candidate source. The candidate sources were compared to the ECD localizations and resection areas.

**Results:**

The kurtosis beamformer produced interpretable localizations in 18/22 patients, of which the candidate source coincided with the resection lobe in 9/13 seizure-free patients and in 3/5 patients with persistent seizures. The sublobar accuracy of the kurtosis beamformer with respect to the resection zone was higher than ECD (56% and 50%, respectively), however, ECD resulted in a higher lobar accuracy (75%, 67%).

**Conclusions:**

Kurtosis beamforming may provide additional value when spikes are not clearly discernible on the sensors and support ECD localizations when dipoles are scattered.

**Significance:**

Kurtosis beamforming should be integrated with existing clinical protocols to assist in localizing the epileptogenic zone.

## Highlights

• Objective localizations of interictal spikes using a kurtosis beamformer.
• Kurtosis Beamforming can provide confidence to scattered dipoles.
• Kurtosis beamforming can assist in localizing the epileptogenic zone.

## 1 Introduction

The aim of epilepsy surgery is to remove the epileptogenic zone (EZ), i.e. the region whose removal ensures postoperative seizure freedom (Engel, 1996, Luders et al., 2006). Hypotheses about the location of the EZ are typically generated on the basis of the patient’s clinical history, as well as electroencephalography (EEG), neuropsychological and neuroimaging assessments (Engel, 1996, Luders et al., 2006, Dorfer et al., 2015). Approximately 15–25% of patients yield inconclusive or non-localizing results (Zumsteg and Wieser, 2000, Carrette et al., 2010) often meaning that additional invasive testing is required (Blount et al., 2008). Magnetoencephalography (MEG) has shown to non-invasively provide unique information to help guide the placement of intracranial electroencephalography (iEEG) electrodes and inform surgical intervention (Mamelak et al., 2002, Fischer et al., 2005, Knowlton, 2006, Sutherling et al., 2008, Stefan et al., 2011, Agirre-Arrizubieta et al., 2014, Nissen et al., 2016).

Clinical MEG analysis usually relies on equivalent current dipole (ECD) fitting to identify sources of interictal paroxysmal abnormalities (spikes) (Ebersole, 1997, Wheless et al., 1999, Bagic et al., 2011). Alternatively, a number of MEG centres have utilised kurtosis beamforming (SAM(g2)) (Robinson et al., 2004, Kirsch et al., 2006, Ishii et al., 2008, Westmijse et al., 2009, Schwartz et al., 2010, Gooijer- van de Groep et al., 2013, Prendergast et al., 2013, Rose et al., 2013, Foley et al., 2014, Wu et al., 2014), a spatial filtering approach that estimates the kurtosis of each region’s time series in source space (Robinson et al., 2004, Kirsch et al., 2006). The underlying hypothesis is that regions containing spikes will have increased kurtosis values relative to regions with normal brain activity. Studies evaluating this method have demonstrated a good level of concordance with other inverse models (Kirsch et al., 2006, Gooijer- van de Groep et al., 2013) and seizure onset zones identified by iEEG (Gooijer- van de Groep et al., 2013, Rose et al., 2013).

Despite a growing body of research, the use of kurtosis beamforming in the clinical analysis of MEG data is variable across sites (Scott et al., 2016) and there are a limited number of studies that have measured its concordance with subsequently resected areas (Guggisberg et al., 2008, Zhang et al., 2011, Tenney et al., 2014). Furthermore, published findings have shown poorer performances relative to ECD fitting (Guggisberg et al., 2008) and reports have suggested it is a time-consuming, cumbersome method (Wu et al., 2014). This may raise scepticism regarding its suitability in the clinical routine procedure (Guggisberg et al., 2008). Therefore, there is a need to further evaluate this approach, particularly in challenging patient samples in whom spiking activity is equivocal.

The perceived rationale for the use of kurtosis beamforming is that it may overcome or assist in reducing the number of subjective steps in the clinical analysis of continuous MEG data, including; (1) the time required to visually inspect 250+ sensor time series (Ishii et al., 2008), (2) the expertise required for the identification of suitable spikes, time points, baseline periods and montages for modeling (Knowlton and Shih, 2004, Bagic et al., 2011, Scott et al., 2016), (3) *a priori* knowledge regarding the number of sources (Gaetz and Cheyne, 2003) and (4) the dipole model to be used (e.g. stationary, rotating, or moving dipole) (Russo et al., 2016). Critically, beamforming allows virtual electrodes to be computed, revealing the time series for predefined locations in the head. The ability to place virtual electrodes at the locations of probable sources of epileptiform activity may assist in detecting transients that are not clearly discernible on the physical sensors (Hillebrand et al., 2016).

In this study we aim to further elucidate the role of kurtosis beamforming in clinical MEG by reporting on its ability to localize the epileptogenic zone in a heterogeneous patient cohort. The patients investigated had varying spike frequencies and inconclusive or conflicting MRI and EEG findings prior to MEG referral. A further aim was to compare the kurtosis beamformer to the original MEG analysis, performed using ECD fitting, and to draw conclusions regarding its added value in generating hypotheses regarding the EZ.

## 2 Methods

### 2.1 Patients

We retrospectively analysed MEG recordings of 22 patients with drug resistant epilepsy as described in (Nissen et al., 2017). The patients underwent preoperative evaluation and epilepsy surgery at the VU University Medical Center, Amsterdam, The Netherlands. Surgery outcome was classified more than 12 months after surgery using the Engel classification. As the patients only underwent routine clinical care, approval for this study and informed consent was not needed by the institutional review board and conformed with the Dutch health law of February 26, 1998 (amended March 1, 2006), i.e. Wet Medisch-Wetenschappelijk Onderzoek met mensen (WMO; Medical Research Involving Human Subjects Act), division 1, Section 1.2.

### 2.2 MEG acquisition

Whole-head MEG recordings were made using an Elekta Neuromag Vectorview system (Elekta Neuromag Oy, Helsinki, Finland) with 306 channels (102 magnetometers and 204 gradiometers) in a magnetically shielded room (Vacuumschmelze GmbH, Hanau, Germany). The acquisition protocol is described in (Nissen et al., 2017) and summarised here: eyes-closed resting-state recordings of 15 min were obtained in the supine position with a 1250 Hz sampling frequency and online filtering (410 Hz anti-aliasing filter and 0.1 Hz high-pass filter). A 3D head-digitizer (Fastrak, Polhemus, Colchester, VT, USA) was used to record the scalp outline and digitize the fiducial landmarks and continuous head position indicator coils. The scalp surface points were co-registered with a T1-weighted MRI of the patient using a surface-matching algorithm using a similar approach as (Adjamian et al., 2004) with an estimated accuracy of 4 mm (Whalen et al., 2008).

### 2.3 Preprocessing

The raw data were spatially filtered offline to remove artefacts using the temporal extension of signal space separation (tSSS) (Taulu and Simola, 2006). This was implemented in the MaxFilter software using a sliding windows of 10 s and a subspace correlation limit of 0.9 (Maxfilter version 2.1, Elekta Neuromag Oy). Noisy channels were visually identified and excluded before tSSS filtering. A single sphere head model was generated based on the co-registered MRI scalp surface and used in both source reconstruction approaches.

### 2.4 ECD analysis

The clinical analysis had already been performed by an experienced EEG/MEG technician. The ECD approach used was consistent with the ACMEGS guidelines (Bagic et al., 2011). In summary, spikes in the sensor time series were identified and a single equivalent current dipole model was calculated at each sample from half-way up the ascending limb of the spike until the peak (using Xfit, version 5.5.18, Elekta Neuromag Oy). Typically, ECD models with goodness of fit (GOF) values above 70% were accepted for further review and were evaluated by a multidisciplinary team of clinicians, physicists and technicians.

### 2.5 Kurtosis beamformer

The kurtosis beamformer was applied to the presurgical MEG data using the Elekta SSS-Spikiness Beamformer (Beamformer version 2.0, Elekta Neuromag Oy). The SSS-beamformer differs from a conventional beamformer in that it operates on the harmonic function amplitudes and the corresponding lead fields derived from SSS filtering (Vrba et al., 2010). The kurtosis beamformer works by reconstructing the source time series for each voxel in the source space grid and then computing the kurtosis value for each of these time series. This results in a volumetric map whereby each voxel is represented by a single kurtosis value. A guide on how to replicate the analysis detailed in this section and an example dataset can be found here: https://osf.io/95k8f/.

To ensure that each dataset underwent the same method, a 300 s time window was chosen for analysis. This time window was selected to include as many spikes as possible whilst trying to avoid artefacts. Data were band-pass filtered from 20 to 70 Hz to provide an optimal contrast for spike identification (Kirsch et al., 2006, Ishii et al., 2008). For each patient, the source space grid (5 mm resolution) was computed for a bounding box enclosing the entire head. Beamformer weights were then constructed and virtual electrodes representing each location in source space were computed. The excess kurtosis (g2) value was then calculated for each virtual electrode time series:

$${\text{g}}_{2}=\frac{\Sigma_{k}^{N}{\left(t(k)-\mu_{t}\right)}^{4}}{N\sigma_{t}^{4}}-3$$

where *N* is the length of time series *t*, μ is the mean and σ is the standard deviation. The volumetric image was then overlaid onto the co-registered MRI and kurtosis peak locations were extracted using a local maxima algorithm in the MRIView software (MRIView version 1.0, Elekta Neuromag Oy). Virtual electrode time series corresponding to the peak locations were recomputed using the stored beamformer weights and compared to the physical sensor time series.

We considered all peaks that were localized inside the head. From these peak locations, the corresponding virtual electrodes were visually inspected to evaluate whether they contained genuine spikes or artefacts. A montage in the Graph software (Elekta Neuromag, Oy) was used to visualize the virtual electrodes alongside the physical MEG sensor time series in 10 s segments. This montage allowed the cross-validation of transients seen in the virtual electrode with those seen in the physical MEG sensors. Virtual Electrodes that robustly localized epileptiform activity (e.g. spikes present in the time series for that location) were selected as a candidate source and included in the analysis. The virtual electrode number chosen as the candidate source is reported in Table 1 (e.g. VE1 represents the first volumetric peak location). To test the value of the kurtosis beamformer in a non-hypothesis driven scenario, no other information (e.g. patient notes, surgical site, EEG, MRI) was used to guide the analysis.

**Table 1:** Patient characteristics, MRI findings, number of spikes in the MEG recording, kurtosis beamformer and ECD localisation, location of the resection and surgery outcome (Engel class) are displayed for all patients. The kurtosis beamformer candidate source location is shown under ‘Kurtosis beamformer localisation’ and the VE peak number is shown under ‘Kurtosis beamformer notes’ (e.g. VE1 represents the first peak location).

*N*	Gender/Age	Interictal EEG	MRI	Spikes in recording	Kurtosis beamformer localisation	Kurtosis beamformer notes	ECD localisation	Resection	Outcome
1	F/25	R temporal	Negative	9	–	No VE candidate	L temporal (cluster)	R temporal	4A
2	F/29	L frontotemporal	MTS L	13	R parietal	VE4 best candidate	L temporal (scatter)	L temporal	1A
3	M/29	R frontal and central	Tumor RI	9	R frontal	VE1 best candidate	R frontobasal (anterior tumor boundary) (scatter)	R Frontal / Insular	3A
4	M/52	–	Tumor L frontal	2	–	No VE candidate	L frontal next to resection cavity (cluster)	L temporal	4B
5	F/46	–	Tumor L frontal	No spikes	–	–	–	L frontal	4B
6	F/26	R neocortical posterior temporal	Tumor R temporal	4	–	Artefacts / No VE candidate	R central (cluster)	R temporal	1A
7	M/28	L frontotemporal	Tumor L frontal	6	L frontal	VE4 best candidate	L frontal (scatter)	L frontal	1A
8	M/40	–	Tumor RF (extends to LF)	No spikes	L central	VE1 best candidate	L central (cluster)	R frontal	4C
9	M/23	L temporal	Tumor L temporal	16	R Frontal	VE2 best candidate	L central (cluster)	L temporal	1A
10	F/33	L neocortical fronto- and medial temporal	Mesial Temporal Sclerosis L	8	L temporal	VE2 best candidates	L temporoparietal (cluster)	L temporal	1A
11	F/52	L > R frontotemporal	Mesial Temporal Sclerosis L	4	L temporal	VE1 best candidate	–	L temporal	1A
12	F/43	R and L frontotemporal	Mesial Temporal Sclerosis R	12	R frontal	VE1 best candidate	R neocortical temporoparietal (cluster)	R temporal	1A
13	M/20	R frontal	Dysplasia R frontal	113	R frontal	VE2 best candidate	R frontal and R temporal (scatter)	R frontal	1A
14	F/29	R > L frontotemporal	Optic tumor	85	R temporal	VE1 best candidate	R medial temporal (cluster)	R temporal	1A
15	F/48	L neocortical medial and posterior temporal	Resection L temporal	9	L temporal	VE9 best candidate	L neocortical temporal (cluster)	L temporal	1A
16	F/33	–	Tumor L temporal	16	R temporal	VE3 best candidate	L temporal behind lesion (cluster) and R temporal (cluster)	L frontal	3B
17	M/38	L > R neocortical frontotemporal	Negative	4	R frontal	VE1 best candidate	L centroparietal (cluster)	L temporal	2A
18	M/47	L frontotemporal	Mesial Temporal Sclerosis L	215	L temporal	VE1 best candidate	L temporal (cluster)	L temporal	1A
19	F/28	L > R temporal	Multiple cavernomas	12	L parietal	VE3 best candidate	L temporoparietal (scatter)	L temporal	1A
20	F/30	L and R frontotemporal	Dysplasia R frontal	12	R frontal	VE1 best candidate	Frontocentral, lateralization not possible (scatter)	R frontal	1A
21	M/39	Frontotemporal, lateralization not possible	Bleeding R temporal + frontal	19	R temporal	VE1 best candidate	R temporal (cluster)	R temporal	2A
22	M/52	L > R frontotemporal	Mesial Temporal Sclerosis L	8	L temporal	VE5 best candidate	L Frontal (cluster)	L temporal	1A

Abbreviations: N: patient number, ECD: equivalent current dipoles, F: female, M: male, L: left, R: right, VE: virtual electrode, –: uninterpretable localisation.

### 2.6 Resection cavity delineation

We manually segmented the resection cavity based on the three month post-operative MRI scan using iPlan 3.0 software (BrainLAB AG, Feldkirchen, Germany). Firstly, the post-operative scan was linearly registered with the preoperative MRI (the one used for MEG co-registration). Secondly, the same transformation that was applied to co-register the preoperative MRI with the MEG data was also applied to the resection cavity.

### 2.7 Concordance with resection cavity

For each patient, the ECD point sources and the single kurtosis beamformer candidate (point) source were overlaid onto the presurgical MRI along with the resection cavity delineation. The ECD results were represented by the cluster or main cluster if the ECDs were scattered. If ECDs were scattered across one lobe, then we regarded the centre of the scatter as the main cluster for determining the overlap. In case of more than one ECD localization, all localizations were reported and considered. If no spikes or focal slow activity were present, or the ECDs extended across multiple lobes, then this was considered as an uninterpretable localization. The kurtosis beamformer results were represented by the location of the candidate source. An uninterpretable kurtosis beamformer localization consisted of virtual electrodes not containing any epileptiform activity. Only interpretable results were included in the concordance calculations.

Anatomical concordance was visually assessed based on the overlap of the kurtosis beamformer candidate source, ECDs and the resection cavity. The level of concordance was determined using concordance criteria similar to that used in Kirsch et al., 2006:

I. Concordant, direct overlap of Kurtosis/ECD and resection: Kurtosis beamformer peak/ECD cluster and resection cavity directly overlap.
II. Concordant, partial overlap of Kurtosis/ECD and resection: Kurtosis beamformer peak/ECD cluster and resected cavity are concordant at the lobar level, but do not directly overlap.
III. Discordant, no overlap of Kurtosis/ECD and resection: Kurtosis beamformer/ECD results were uninterpretable or disagreed on location with resection cavity (e.g. scattered ECD results).

### 2.8 Concordance between ECD and kurtosis beamformer localizations

The overlay of the ECD point sources and the single kurtosis beamformer candidate source were used to establish concordance in a similar manner as described in the paragraph above:

I. Concordant, direct overlap of Kurtosis and ECDs: Kurtosis beamformer peak and ECD main cluster directly overlap.
II. Concordant, partial overlap of Kurtosis and ECDs: Kurtosis beamformer peak and ECD main cluster are contained in the same lobe, but do not directly overlap.
III. Discordant, no overlap of Kurtosis and ECDs: Kurtosis beamformer peaks and ECD main cluster are in different lobes.

### 2.9 Sensitivity, specificity, and accuracy

To evaluate the concordance between the two source localization methods and the resection cavity, measures of sensitivity, specificity and accuracy were calculated regarding the surgery outcome. These measures were calculated only on the interpretable localizations (e.g. non-localizing ECD scatters, kurtosis peaks outside of the head were not included). Sensitivity was based on the number of ECD/Kurtosis beamformer localizations that overlapped with the resection cavity in the patients that were seizure free. Specificity was based on the number of discordant ECD/Kurtosis beamformer localization with the resection cavity in patients with persistent seizures. More specifically:

Sensitivity = Concordance with resection area in seizure-free patients / all seizure-free patients.
Specificity = Discordance with resection area in patients with persistent seizures / all patients with persistent seizures.
Accuracy = (Concordance with resection area in seizure-free patients + discordance with resection area in patients with persistent seizures) / all patients.

Furthermore, the difference in accuracy (overlap with resection area in seizure-free patients and non-overlap in patients with persistent seizures) between the two methods was tested at the lobar and sublobar level using a chi-square test for inhomogeneity.

## 3 Results

Presurgical MEG data from 22 patients who subsequently had a focal cortical resection were retrospectively analysed using a kurtosis beamforming approach. The presurgical findings from before the MEG referral of the patients were inconclusive or conflicting, and are displayed alongside patient characteristics in Table 1. The number of spikes present in the MEG recording differed from no spikes (two recordings) to 215 spikes (median: 9 spikes). The kurtosis beamformer resulted in a localization in 18/22 patients (82%). Of the four patients with an uninterpretable kurtosis beamformer localization; one had no spikes in the MEG recording (patient 5), one had no spikes visible on the virtual electrodes (patient 4), and two had excessive artefacts in their recording so that the kurtosis beamformer peaks were either outside the head (patient 6) or the virtual electrodes showed only artefacts (patient 1). The ECD analysis localized in 20/22 patients (91%), either in a delimited area (cluster in 14 patients) or widespread (scatter in 6 patients).

**Fig. 1:**
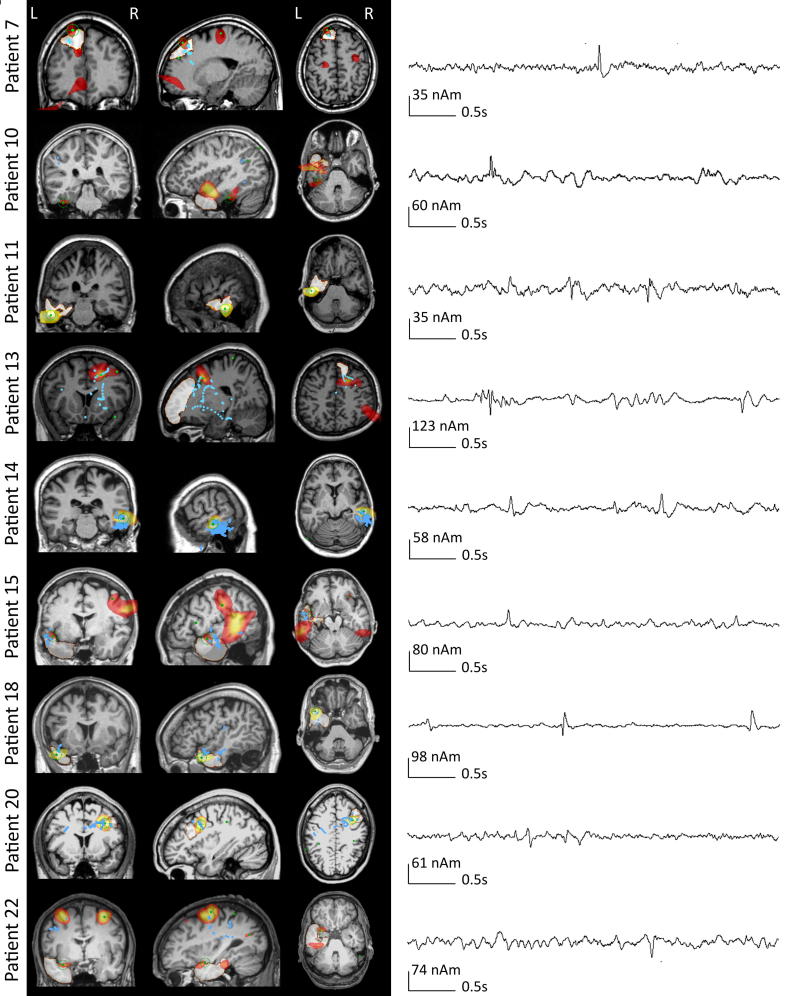
Examples of all seizure-free patients, in whom the kurtosis beamformer results were concordant (sublobar and lobar overlap) with the resection cavity. **Left**: the preoperative structural MRI is shown in three views with overlays of the resection area (milky area), kurtosis beamformer results (hot/orange), placement of the VE in the kurtosis peaks (green dots), and ECD location (blue dots). The empty green circle centres on the best VE candidate for the kurtosis beamformer results. Slice views are centred around the kurtosis beamformer candidate source, therefore not all ECD point sources are visible. **Right**: A four second segment of the virtual electrode time series corresponding to the candidate source (the virtual electrode chosen as the kurtosis beamformer localisation) for each patient.

Table 2 shows the concordance of the kurtosis beamformer localization with the resection cavity and ECD localization. For the seizure-free patients, in whom the resection cavity corresponds to the epileptogenic zone, the kurtosis beamformer overlapped with the resection cavity in 9/13 patients (69%) (6 direct overlap and 3 partial overlap). Fig. 1 shows the localization results and virtual electrode time series for these nine patients. In the patients with persistent seizures (i.e. the epileptogenic zone was not entirely removed or disconnected), the kurtosis beamformer was concordant with the resection cavity in 2/5 patients (40%) (1 direct overlap and 1 partial overlap). In summary, the kurtosis beamformer mainly localized to the resection cavity in seizure-free patients but not in patients with persistent seizures. Table 3 shows the sensitivity (regarding overlap in seizure-free patients), specificity (regarding discordance in patients with persistent seizures), and accuracy (regarding all correct concordances and discordances). The accuracy was 56% on a sublobar level (direct overlap) and 67% on a lobar level (direct and partial overlap) for the kurtosis beamformer.

**Table 2:** Concordance between kurtosis beamformer localisation, resection cavity, and ECD localisation. Surgery outcome is provided in Engel classes.

Patient	Surgery outcome	Concordance Kurtosis/resection	Concordance ECD/resection	Concordance Kurtosis/ECD
*Seizure-free patients*				
2	1A	Discordant	Concordant, partial overlap	Discordant
6	1A	–	Discordant	–
7	1A	Concordant, direct overlap	Concordant, direct overlap	Concordant, direct overlap
9	1A	Discordant	Discordant	Discordant
10	1A	Concordant, partial overlap	Concordant, partial overlap	Discordant
11	1A	Concordant, direct overlap	–	–
12	1A	Discordant	Concordant, partial overlap	Discordant
13	1A	Concordant, partial overlap	Concordant, direct overlap	Concordant, direct overlap
14	1A	Concordant, partial overlap	Concordant, partial overlap	Concordant, direct overlap
15	1A	Concordant, direct overlap	Concordant, direct overlap	Concordant, direct overlap
18	1A	Concordant, direct overlap	Concordant, direct overlap	Concordant, direct overlap
19	1A	Discordant	Concordant, partial overlap	Concordant, partial overlap
20	1A	Concordant, direct overlap	Concordant, direct overlap	Concordant, direct overlap
22	1A	Concordant, direct overlap	Discordant	Discordant

*Patients with persistent seizures*				
1	4A	–	Discordant	–
3	3A	Concordant, partial overlap	Concordant, direct overlap	Concordant, partial overlap
4	4B	–	Discordant	–
5	4B	–	–	–
8	4C	Discordant	Discordant	Concordant, direct overlap
16	3B	Discordant	Discordant	Concordant, direct overlap
17	2A	Discordant	Discordant	Discordant
21	2A	Concordant, direct overlap	Concordant, direct overlap	Concordant, direct overlap

Abbreviations: ECD: equivalent current dipoles, –: uninterpretable localisation.

**Table 3:** Sensitivity, specificity and accuracy for the concordance between kurtosis beamformer localisation, resection cavity, and ECD localisation.

	Concordance Kurtosis/resection		Concordance ECD/resection		Concordance Kurtosis/ECD*	
	Direct overlap (sublobar concordance)	Partial + direct overlap (lobar concordance)	Direct overlap (sublobar concordance)	Partial + direct overlap (lobar concordance)	Direct overlap (sublobar concordance)	Partial + direct overlap (lobar concordance)
Seizure-free patients	6/13	9/13	5/13	10/13	6/12	7/12
Patients with persistent seizures	1/5	2/5	2/7	2/7	3/5	4/5
Total	7/18	11/18	7/20	12/20	9/17	11/17
Sensitivity	46%	69%	38%	77%		
Specificity	80%	60%	71%	71%		
Accuracy	56%	67%	50%	75%		

Abbreviation: ECD: equivalent current dipoles.

* For the concordance between kurtosis beamforming and ECD analysis, the resection area and surgery outcome was not taken into account, hence sensitivity, specificity and accuracy could not be calculated.

ECD localizations were concordant with the resection cavity in 10/13 seizure-free patients (77%) (5 direct overlap and 5 partial overlap) (Table 2). In patients with persistent seizures, 2/7 patients (29%) (2 direct overlap) had concordant results. The accuracy was lower for the ECD localization (50%) compared to the kurtosis beamformer localization (56%) on a sublobar level, but was higher on a lobar level (75% for ECD analysis and 67% for kurtosis beamformer) (Table 3). However, the differences remained non-significant at both the sublobar (χ^2^ (1) = 0.117, *p* = 0.76) and lobar (χ^2^ (1) = 0.320, *p* = 0.72) level.

Concordances of the two methods were moderate to high regardless of surgery outcome (Table 2). For seizure-free patients with an interpretable localization by both methods, the kurtosis beamformer coincided with ECD localizations in 7/12 patients (58%) (six direct overlap and one partial overlap). In the patients with persistent seizures, the kurtosis beamformer corresponded to the ECD localization in 4/5 patients (80%) (three direct overlap and one partial overlap). In total, the kurtosis beamformer co-localized with the ECD analysis in 9/17 (53%) on a sublobar level and in 11/17 (65%) on a lobar level (Table 3).

The kurtosis beamformer resulted in a more accurate localization than the ECD analysis in six patients. Of these, the kurtosis beamformer candidate source directly overlapped with the resection area in two patients, whereas the ECD localizations were either uninterpretable (patient 11) or localized to another lobe (patient 22). In patients 7, 13 and 20 the ECDs were scattered and fell both inside and outside of the resection area, whereas the kurtosis beamformer produced an unambiguous source (i.e. the virtual electrode showed clear spiking activity). For example, in patient 20, the ECDs were not lateralised, whereas the kurtosis beamformer directly overlapped with the resection area. In a further patient (patient 10), the kurtosis beamformer candidate source was adjacent to the resection area in the anterior temporal lobe, whereas the ECDs localized to a more posterior area near the temporal-parietal junction.

In patients 2, 12 and 19, the kurtosis beamformer candidate source was discordant with the resection cavity, whereas the ECD localizations partially overlapped. These patients did produce kurtosis beamformer peaks in areas concordant with the resection area but based on our inspection of the virtual electrode time series an alternative candidate source was selected. In patient 13, the ECD localization directly overlapped with the resection cavity whereas the kurtosis beamformer only partially overlapped.

## 4 Discussion

The purpose of this study was to test the performance of the kurtosis beamformer in a heterogeneous group of patients with varying spike activity. The kurtosis beamformer candidate sources were compared to the clinical ECD analysis and the resection area in both seizure free and seizure persistent patients. We found that the kurtosis beamformer provided an interpretable localization in the majority of patients (18/22). Of these, the candidate source was contained within the resection lobe in 9/13 seizure-free patients and in 2/5 patients with persistent seizures, yielding an accuracy of 67% on a lobar level. The kurtosis beamformer had a higher accuracy than the ECD analysis on the sublobar level (56% and 50%, respectively) but not on the lobar level (67% and 75%, respectively). However, these differences were not statistically significant.

Previous studies that have evaluated the kurtosis beamformer relative to iEEG found lobar concordance in the majority of patients (e.g. Tenney et al., 2014, Wu et al., 2014, Zhang et al., 2011). It can be suggested that the gold standard for evaluating the performance of a clinical source localization method is by measuring its spatial concordance with the resection area in seizure-free patients (i.e. the EZ). In our study, we aimed to further evaluate kurtosis beamforming by retrospectively comparing its output to the resection area in combination with surgery outcome. Our study found a higher level of concordance between the kurtosis beamformer and the epileptogenic zone (9/13) relative to a similar study by Guggisberg et al. (2008) who reported a concordance of 3/11 in seizure-free patients. A key difference between the two studies is that Guggisberg and colleagues did not visually inspect the virtual electrode time series corresponding to the kurtosis beamformer peaks.

It is important to reiterate the necessity for inspecting the virtual electrode time series to rule out artefacts, to ensure that kurtosis peak locations contain spikes, and to determine the relationship between multiple foci (Rose et al., 2013, Scott et al., 2016). This manual verification step still involves the visual assessment of time series, but only for a small set of virtual electrodes with higher SNR relative to the many (a few hundred) physical sensors. We found this step not to be as extensively time-consuming as previously suggested (Wu et al., 2014). Furthermore, to reduce visual inspection time, a peak-to-root mean square ratio algorithm can be used to automatically mark spikes in the virtual electrode time series (Kirsch et al., 2006).

In our study, visual inspection of the virtual electrodes that corresponded to the volumetric kurtosis peaks inside the head was critical (5–10 peaks per patient). The highest peaks were not necessarily the best candidates and visual inspection helped to identify sources that coincided with the EZ despite the presence of artefacts. Scott et al. (2016) suggested reviewing the first five kurtosis peaks, which may work well for artefact-free MEG recordings. In contrast, our datasets included several recordings with noisy channels and muscle artefacts, despite our efforts to minimise these. This resulted in multiple artefact-driven peaks. Patient compliance is therefore important for limiting excessive or re-occurring physiological artefacts (e.g. jaw clenching) that may bias the kurtosis metric towards spurious sources.

Another goal of this study was to compare the kurtosis beamformer to the original clinical ECD analysis. Overall, the two methods showed a moderate overlap with one another (53% sublobar, 65% lobar), which is consistent with other studies showing similar or higher lobar agreements (Kirsch et al., 2006, Zhang et al., 2011, Wu et al., 2014). The kurtosis beamformer achieved a higher accuracy at the sublobar level, whereas the ECD analysis showed a higher accuracy at the lobar level. Importantly, our findings demonstrated how the kurtosis beamformer can provide additional information to the ECD analysis. In two seizure free patients (11 and 22), the kurtosis beamformer localized sharp atypical activity to the EZ (direct overlap) whereas the ECD analysis resulted in discordant localizations. The clinical value of localizing sharp atypical activity remains to be established, however, the ability to do so may be useful in the absence of clear spikes. In three additional patients (7, 13 and 20), ECD scatters fell both inside and outside of the resection area, whereas the kurtosis beamformer gave an unambiguous localization within the resection area. This suggests that the kurtosis beamformer may instil confidence in the results of ECD analysis, particularly when the ECDs are scattered.

We also found that ECD scatters localized the EZ (partial overlap) in three patients (2, 12, 19), whereas the kurtosis beamformer candidate source did not. In these patients, the kurtosis beamformer produced multiple peaks containing spikes, some of which overlapped with the ECD scatters. Our selection of the candidate source in these patients was based on the source that robustly localized spikes, however these locations were not concordant with the EZ. This reaffirms that interictal spikes are not necessarily an index of the EZ (Lüders et al., 2006) and can occur in distant or contralateral regions (Zumsteg et al., 2005). This finding highlights the need to interpret the kurtosis beamformer in the context of all available clinical information, which we did not do in this study in order to test its performance in an unbiased way. Therefore, interpreting the results in regard to other presurgical information (e.g. MRI, EEG) is recommended to determine whether localized spikes are a probable marker of the EZ or a result of propagation along the neural pathways.

Both kurtosis beamforming and ECD analysis localize spikes and sharp waves, but their method of detection differs. ECD analysis relies on the visual identification of spikes in the physical MEG sensors, which may miss sharp atypical activity or low amplitude activity (as shown in this work). On the other hand, the kurtosis beamformer detects irregularly occurring activity in the source time series. As in any automatic detection method, spikes might be missed or artefacts might obscure spike detection. Our results showed that in some patients one of the methods correctly localized the EZ, whereas the other method failed to do so. These different detection sensitivities make the two methods complementary, and we therefore suggest using both methods for clinical analysis. The initial use of the kurtosis beamformer could be particularly useful in estimating the number of sources for ECD analysis or in cases where ECD analysis does not yield interpretable results.

A limitation of our comparison between the kurtosis beamformer and the ECD analysis was the spatial extent of the localizations: the kurtosis beamformer output was reduced to a single point source, whereas the ECD analysis resulted in multiple point sources that could be clustered or scattered. For example, in patient 13 the kurtosis candidate source was adjacent to the resection area whereas the dipoles fell within and around the resection area, resulting in a partial overlap. Moreover, the choice of location and resolution of the beamformer source grid has limited its spatial resolution to 5 mm. In ‘near miss’ cases, such as patient 13, a higher resolution grid (<5 mm spacing) may have increased the kurtosis beamformer accuracy leading to a direct overlap with the resection area, although this was not tested here. Further increases in accuracy may be limited though, since it has previously been demonstrated that only approximately 10% of the source space might benefit from a grid resolution that is higher than 5 mm (Barnes et al., 2004). Another limitation in comparing the kurtosis beamformer to the resection area is that the resection site may have been influenced by the ECD findings as they formed part of the original presurgical evaluation.

The heterogeneous patient cohort used in this study is representative of the patients typically referred to MEG for presurgical evaluation in our centre. The generalizability of these findings may benefit from larger patient studies whereby kurtosis beamforming can be evaluated in subgroups, such as temporal or frontal lobe epilepsy patients. Open source approaches to computing the kurtosis beamformer are now becoming available (FieldTrip, MNE) and may facilitate larger multicentre studies whereby data from different sites and MEG platforms can be pooled together and analyzed using a standardized set of analysis scripts.

Recently, a sliding SAM(g2) approach (SAMepi) has been proposed in order to maximise the kurtosis value for sources that produce very frequent spikes (Harpaz et al., 2015, Scott et al., 2016). The problem often encountered in our patient population is that patients tend to produce few interictal spikes (Nissen et al., 2016) and may therefore not benefit from this approach. The dependence on spikes is a general limitation of both kurtosis beamforming and ECD analysis, hence alternative methods are needed to generate hypotheses regarding the EZ in the absence of spikes. The placement of virtual electrodes in suspected source locations, for example based on MRI and EEG findings, may assist in this situation (Hillebrand et al., 2016). Furthermore, it has been shown that network analysis can identify the epileptogenic zone in MEG data without interictal spikes (Nissen et al., 2017). Future research should continue to focus on developing and validating methods that detect the full spectrum of epileptiform activity (e.g. high frequency oscillations, spikes, atypical slow waves), as well as investigating spike-independent approaches.

## 5 Conclusions

Our results show that kurtosis beamforming performs comparably to ECD but with fewer subjective steps and without the need of *a priori* information to guide the analysis. Kurtosis beamforming can assist the ECD analysis by instilling confidence in the ECD localizations (particularly when scattered) and in some cases localize unknown or unexpected sources. We propose that kurtosis beamforming should be integrated with existing clinical protocols to assist in generating hypotheses regarding the EZ. This could be achieved with little additional effort by taking the agreement of both approaches (de Gooijer-van de Groep et al., 2013) and in cases where the two methods are discordant, virtual electrodes can be placed in the kurtosis peaks and ECD clusters to determine clinical relevance. Kurtosis beamforming could also be used as a first pass analysis to estimate the number of probable sources to model and to automatically identify spikes in the time series. This may assist in the early subjective steps encountered during ECD analysis.
